# A fully roll-to-roll gravure-printed carbon nanotube-based active matrix for multi-touch sensors

**DOI:** 10.1038/srep17707

**Published:** 2015-12-04

**Authors:** Wookyu Lee, Hyunmo Koo, Junfeng Sun, Jinsoo Noh, Kye-Si Kwon, Chiseon Yeom, Younchang Choi, Kevin Chen, Ali Javey, Gyoujin Cho

**Affiliations:** 1 Regional Innovation Center for Printed Electronics, Sunchon National University, Sunchon 540-742, Korea; 2 Department of Printed Electronics Engineering, Sunchon National University, Sunchon 540-742, Korea; 3 Department of Mechanical Engineering, Soonchunhyang University, Asan 336-745, Korea; 4 Electrical Engineering and Computer Sciences, University of California, Berkeley, California 94720, United States

## Abstract

Roll-to-roll (R2R) printing has been pursued as a commercially viable high-throughput technology to manufacture flexible, disposable, and inexpensive printed electronic devices. However, in recent years, pessimism has prevailed because of the barriers faced when attempting to fabricate and integrate thin film transistors (TFTs) using an R2R printing method. In this paper, we report 20 × 20 active matrices (AMs) based on single-walled carbon nanotubes (SWCNTs) with a resolution of 9.3 points per inch (ppi) resolution, obtained using a fully R2R gravure printing process. By using SWCNTs as the semiconducting layer and poly(ethylene terephthalate) (PET) as the substrate, we have obtained a device yield above 98%, and extracted the key scalability factors required for a feasible R2R gravure manufacturing process. Multi-touch sensor arrays were achieved by laminating a pressure sensitive rubber onto the SWCNT-TFT AM. This R2R gravure printing system overcomes the barriers associated with the registration accuracy of printing each layer and the variation of the threshold voltage (V_th_). By overcoming these barriers, the R2R gravure printing method can be viable as an advanced manufacturing technology, thus enabling the high-throughput production of flexible, disposable, and human-interactive cutting-edge electronic devices based on SWCNT-TFT AMs.

Since its first appearance in 1906^1^, roll-to-roll (R2R) gravure has evolved and is currently the highest throughput printing technique with the highest resolution for printing magazines and packaging^2^. R2R gravure printing is now further considered to be the core of an innovative technology for building flexible, disposable, and human-interactive electronic devices. Because of the robustness of the gravure cylinder^3^ and advances in fine engraving technology that enable fine patterns^4^, this technology has been further developed as an advanced manufacturing method to build various devices, including radio frequency identification (RFID) tags^5^, smart packaging^6^, roll-able signage^7^, and roll-able displays^8^. However, the practical realization of R2R gravure printing for those electronic devices faces many challenges related to the overlay printing registration accuracy (OPRA)^9^, the R2R web handling^10^, and the curing conditions for the electronic inks (<5 s at 150 °C)^11^. These issues must be overcome to achieve continuous printing of thin film transistor (TFT)-based electronic devices with a practical printing speed (>5 m/min) and a practical device yield^12^,^13^. Currently, the OPRA of our R2R gravure, which represents an output value that includes the results of R2R web handling, can reach up to ±20 μm at a poly(ethylene terephthalate) (PET) web transfer speed of 8 m/min. To satisfy this R2R gravure printing speed, the three different types of inks used to print the interconnects and source/drain electrodes, dielectric layers, and active layers should show the required printability on an inexpensive substrate, such as PET or paper, while maintaining the minimum electrical properties to operate the R2R-printed TFT-based electronic devices under 20 V DC with a practical device yield (>90%). To achieve the R2R-printed TFT-based electronic devices, the following issues should be first addressed:

First, all inks must dry within 5 s at 150 °C.

Second, the resistance of the printed electrodes and interconnects should be lower than 4 mΩ/sq/mil.

Third, the gate capacitance of the printed dielectric layers should exceed 7 nF/cm^2^ without any pinhole.

Finally, a charge carrier mobility of the printed semiconducting layer should exceed 0.01 cm^2^/V-s to obtain a reasonable operation current and frequency.

Fully R2R-printed TFT-based functional electronic devices have not been reported previously in the literature. Only R2R gravure-printed rectennas^12^ and wireless RF-sensor labels^13^ have been reported by our group using R2R gravure-printed diodes, capacitors, antennas and electrochromic signages. The major reason for the lack of results related to R2R-printed TFT-based electronic devices results from the difficulties in achieving a high device yield (>90%) and in controlling the threshold voltage (V_th_) variation in R2R-printed TFTs. The proper operation of the electronic devices is directly related to the number of integrated working TFTs and the range of the V_th_ variation (ΔV_th_(t) ~ eN_tr_(t)/C_o_, where t is time, C_o_ is the capacitance and N_tr_ is the trapped charge density)^14^,^15^,^16^. Our group recently reported critical factors of the V_th_ variation in R2R gravure-printed TFTs, with five different channel lengths (CLs, i.e., 30, 80, 130, 180, and 230 μm) using a single-walled carbon nanotube (SWCNT)-based ink as an active ink and the range of the V_th_ variation with the CL of 130 μm was shown to be 34% with a 100% device yield^17^ on a 150 m PET roll. Integrating any practical logic circuit using R2R gravure remains difficult because of the large V_th_ variation ( ± 0.62 V). However, R2R gravure can be utilized to print SWCNT based TFT (SWCNT-TFT) active matrices (AMs) that can be applied as the backplane for controlling sensor arrays and displays, which do not require such a narrow range in the V_th_ variation. The SWCNT-TFT-based AMs can be properly operated with up to 100% V_th_ variation^18^,^19^, and favor the R2R gravure manufacturing process to build flexible, inexpensive, and arbitrarily large-area AMs (>1 × 10 m^2^).

In this paper, as a tactical strategy for demonstrating the first step in how to reach fully R2R gravure-printed SWCNT-TFT based AMs for signage (<40% of V_th_ variation) and displays (<10% of V_th_ variation), 20 × 20 SWCNT-TFT based AMs with a 9.3 ppi resolution are investigated (Fig. 1). The PET roll, silver-nanoparticle-based conducting ink, SWCNT-based semiconducting ink, and BaTiO_3_-nanoparticle-based dielectric ink are employed in this work. The resultant fully R2R gravure-printed AM is then demonstrated as a pressure sensor matrix for potential applications in multi-touch sensor arrays by laminating a pressure-sensitive rubber sheet.

## Results

Throughout this study, the effects of the OPRA of the R2R gravure machine and the CL of the SWCNT-TFTs on the device yield and V_th_ variation of the SWCNT-TFTs are explored. The R2R gravure printing setup is as follows: a 15 × 0.25 m^2^ PET roll with a thickness of 100 μm as the web, a printing speed of 8 m/min, an impression roller pressure of 6 kg_f_, a web tension of 5 kg_f_, a 40° contact angle between the blade and gravure cylinder, and pyramidal engraved cell structures on the gravure cylinder (10–35 μm in depth and 55–130 μm in width as illustrated in Supplementary Fig. S1). The engraved cell structures are selected based on our previous results from R2R gravure-printed SWCNT-TFTs on a 150 m PET roll^17^. Further details of the R2R gravure printing condition are summarized in Supplementary Table S1.

**Figure 1:**
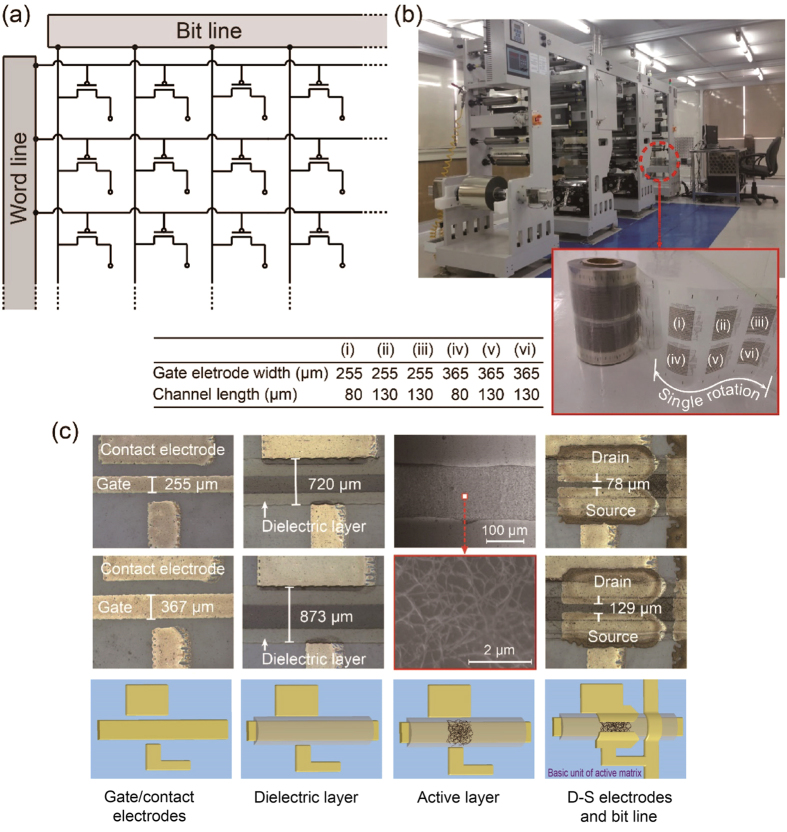
R2R gravure to fully print 20 × 20 TFT AMs on a PET roll. (**a**) Circuit layout of the 20 × 20 TFT AMs and (**b**) optical image of the R2R gravure with an inset image of the fully R2R gravure printed 20 × 20 TFT AMs on a PET roll showing six AMs with different CLs and GWs (i–vi), printed via a single rotation of the gravure cylinder. (**c**) Optical images with descriptive schematic drawings of the printed gate electrode, dielectric layer, active layer (SEM image of the SWCNT layer) and source/drain electrodes with a bit line which display a basic unit of TFT-active matrix.

**Figure 2:**
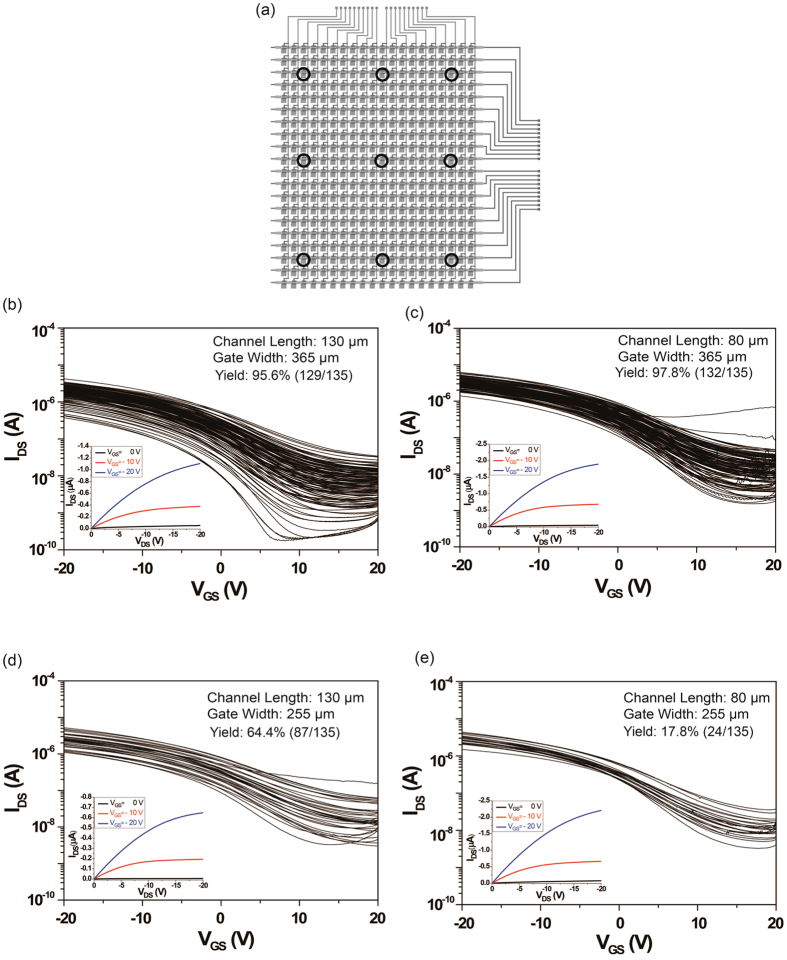
The statistical transfer characteristics of SWCNT-TFTs on the 15 m PET web. (**a**) Schematic of a 20 × 20 TFT AM array indicating the selected TFTs measured at each 1 m along the 15 m PET web. The combined output transfer characteristics of the selected TFTs for AMs with CLs of (**b**) 130 μm and (**c**) 80 μm and GWs of 365 μm as well as TFTs with channel lengths of (**d**) 130 μm and (**e**) 80 μm with 255 μm gate electrode widths of 255 μm. The typical output characteristic of printed TFT was shown in the inset of each graph for the transfer characteristics.

The R2R gravure with two printing units, manufactured by i-Pen Korea, was used with a custom servomechanism system (see Supplementary Fig. S2 for details) in a class 10,000 clean room with the humidity and temperature controlled to 40 ± 2% and 23 ± 1 °C, respectively. Because four printing units are required to complete the TFT-based active matrix, we first start printing the first metal layer consisting of the gate electrodes (widths of 255 and 365 μm), the bus lines (widths of 255 and 365 μm), and the contact electrodes (860 × 760 μm^2^) at a printing speed of 8 m/min with the R2R gravure. Then, we continue to print the dielectric layer (widths of 720 and 875 μm) on the second printing unit with the same printing speed. The roll is rewound after the completion of the dielectric layers. Then, the active SWCNT layer is printed on the second printing unit. Finally, the roll is rewound again and the source-drain electrodes are printed with CLs of 80 and 130 μm and a width of 1420 μm. All electronic inks (silver-nanoparticle-based ink, BaTiO_3_-nanoparticle-based ink, and SWCNT-based ink) were purchased from PARU Co. Korea, and their rheological characteristics were fine-tuned to satisfy the printing conditions (Supplementary Table S2). The gate electrode widths (GWs) are designed to be larger than the CLs to allow alignment tolerances resulting from the limit of the OPRA and to yield lower typical resistance (2.5 mΩ/sq/mil). An optical image of the R2R gravure and the printed SWCNT-TFT AM on a PET roll is shown in Fig. 1b. In total, 220 AMs are printed on each 15 m roll of PET. Six 20 × 20 SWCNT-TFT AM can be printed by a single rotation of the gravure cylinder with two different GWs (255 and 365 μm) and two different CLs (80 and 130 μm) (see the inset image of Fig. 1b). The stepwise illustrations for printing the gate electrode, dielectric layer, active (SWCNT) layer, and drain-source electrodes with real printed images are shown in Fig. 1c. Although the real physical dimensions of the printed gate electrodes (255 and 367 μm), dielectric layers (720 and 873 μm) and drain-source electrodes (CLs of 78 and 129 μm) are slightly different from the designed dimensions (Fig. 1c), all vary by less than 3%. For the convenience of extracting the scalability factors, we characterised 9 TFTs per AM (consisting of 400 TFTs each) along the 15 m of the PET roll (Fig. 2a).

**Figure 3:**
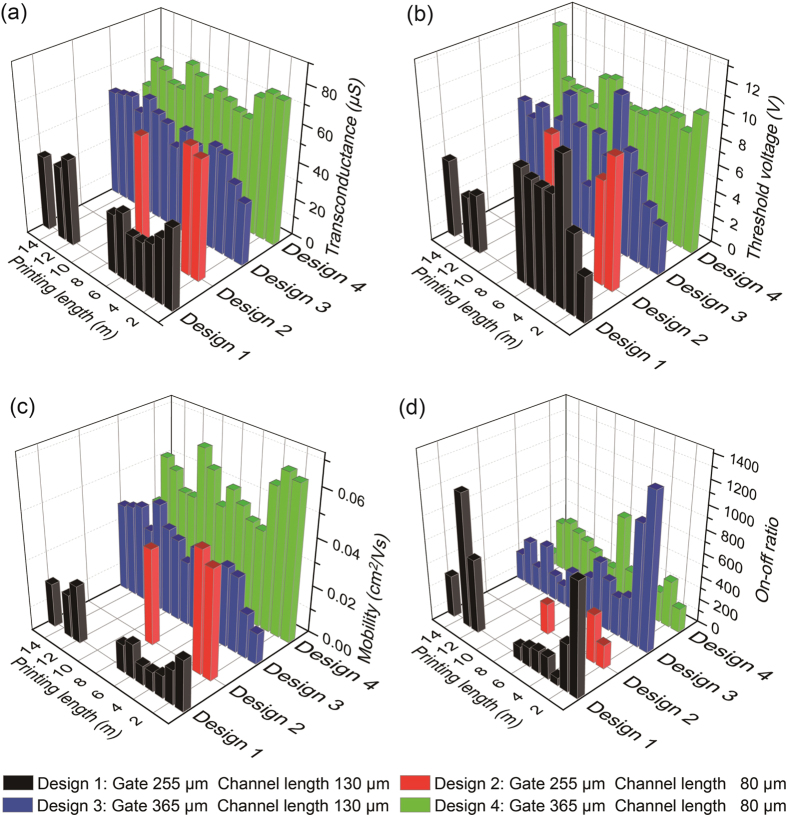
Bar graphs showing the average extracted electrical parameters of SWCNT-TFTs with different CLs and GWs along each 1 m of a 15 m PET roll. (**a**) Transconductance, (**b**) threshold voltage, (**c**) mobility and (**d**) on-off current ratio.

The transfer characteristics and device yield of gravure-printed 20 × 20 SWCNT-TFT AMs are shown in Fig. 2b–e respectively for CLs of 80 μm and 130 μm, GWs of 255 μm and 365 μm, and a channel width of 1420 μm. For the AMs with a CL of 80 μm and a GW of 365 μm, the device yield is approximately 97.8% with a V_th_ variation of 9.18 ± 1.74 V. The device yield was calculated without any short or open SWCNT-TFTs. All failures originated from the short SWCNT-TFTs. The AMs with a CL of 80 μm and a GW of 255 μm display a low device yield (17.8%) with a V_th_ of 8.88 ± 1.22 V. The transfer characteristics of the selected SWCNT-TFTs in each printed 20 × 20 SWCNT-TFT AM at every 1 m along the 15 m PET roll are presented in Supplementary Figs S3 and S4. For every rotation of the gravure cylinder, six of the 20 × 20 SWCNT-TFT-based AMs are printed (Fig. 1b). Among the six AMs printed per rotation, the device yields and V_th_ variation are largely constant. Lower device yields are observed for narrow gate electrodes (a width of 255 μm) because of the misalignment of the source-drain electrodes to the gate electrodes resulting from the limits of OPRA (Fig. 2d,e). Aside from the OPRA, the lower device yield for the samples with a CL of 80 μm and a GW of 255 μm can also originate from periodic irregularities in the circumference of the gravure cylinder roll and the deformation of the PET film. The extracted average charge carrier mobility, on–off current ratio, V_th_ and transconductance for 9 random TFTs at each 1 m along a 15 m PET roll are measured and presented in Fig. 3. Only a small fluctuation of the extracted parameters occurs for the devices with a GW of 365 μm because the CLs and GWs are well matched to compensate the limits of the OPRA. While the PET passes through four heating chambers at a temperature of 150 °C for 7.5 s, the PET film dominantly expands along the machine direction under the web tension of 5 kg_f_. Therefore, using the given R2R gravure system, an OPRA with a well-matched GW and CL will allow for the manufacturing of the SWCNT-TFT AM with a resolution of 9.3 ppi. The key scalability factor to develop a high device yield (>95%) in this R2R gravure system is used to select the most appropriate CL and GW in conjunction with the OPRA. This selection represents the “design rules” for our R2R gravure technology.

**Figure 4:**
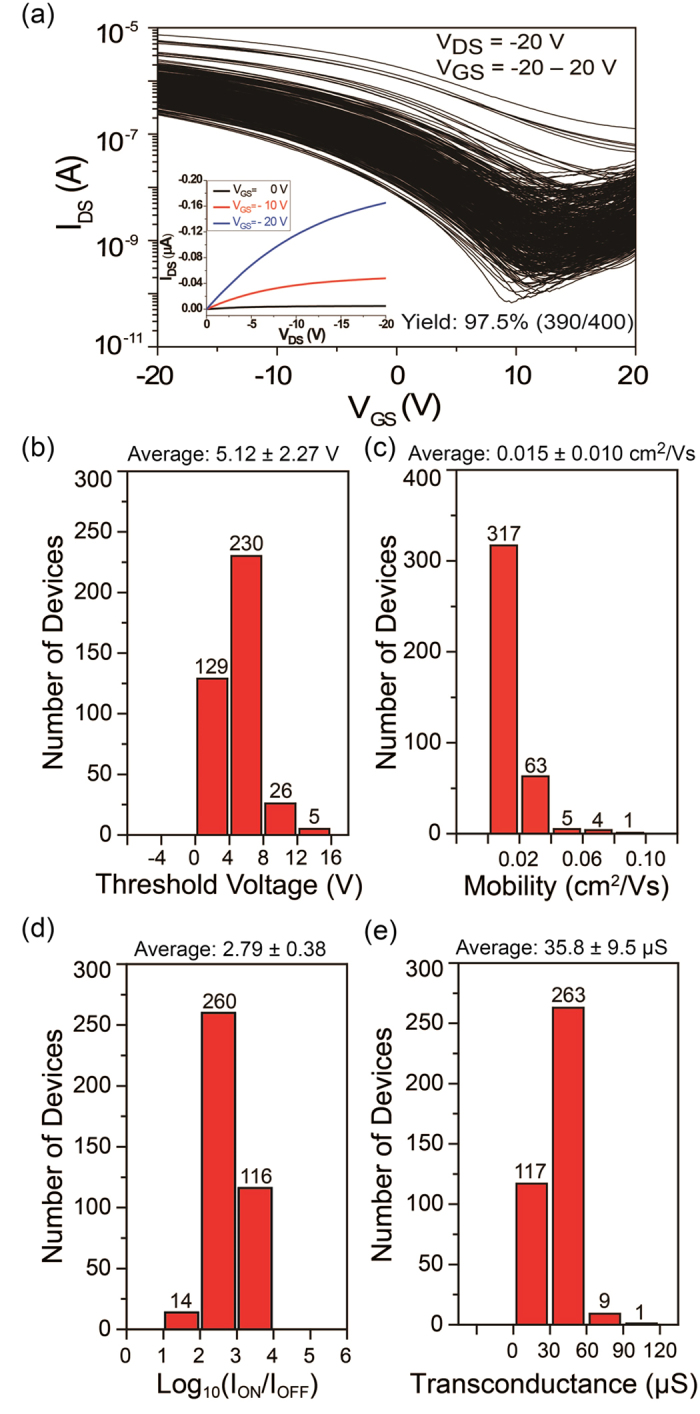
Electrical characteristics of a randomly selected 20 × 20 SWCNT-TFT AM with a CL of 80 μm and a GW of 365 μm to fully analyse the electrical characteristics. (**a**) Transfer characteristics of 400 TFTs and a typical output characteristic in the inset, (**b**) threshold voltage distribution, (**c**) mobility distribution, (**d**) log on-off current ratio distribution and (**e**) transconductance distribution.

**Figure 5:**
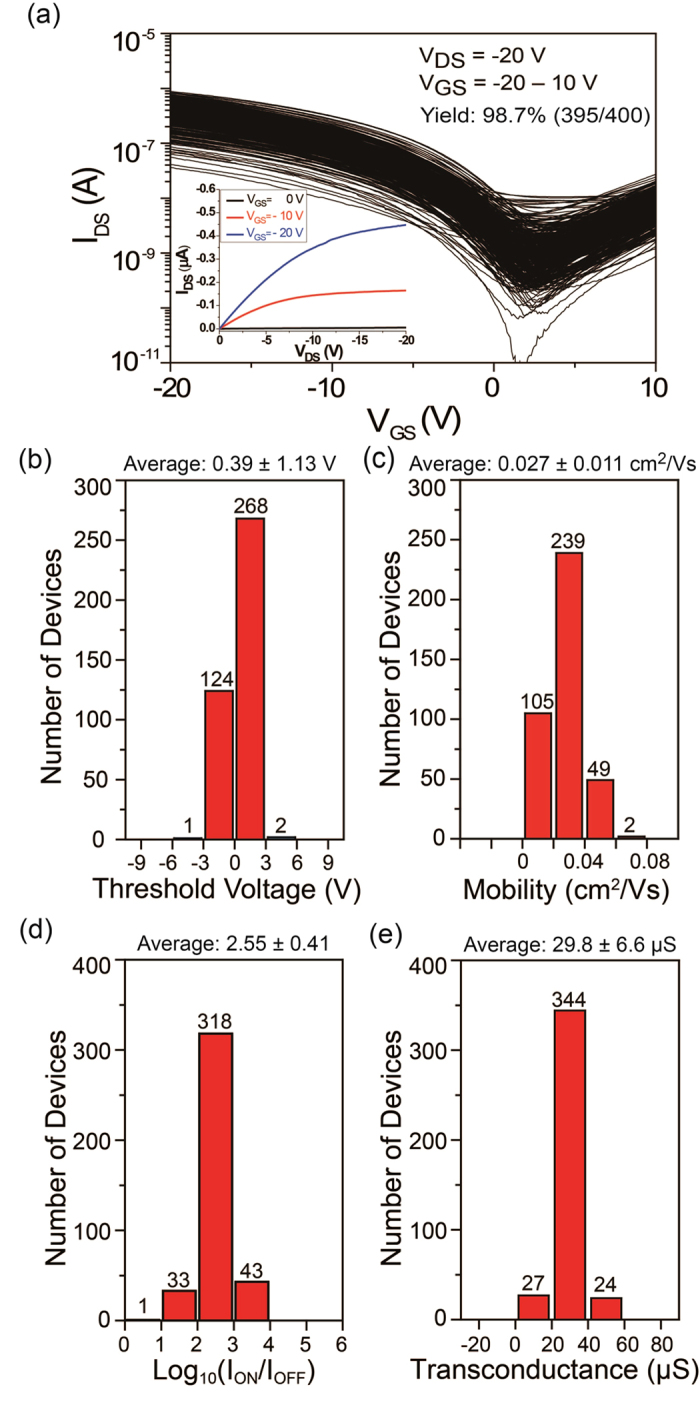
Electrical characteristics of a randomly selected 20 × 20 SWCNT-TFT AM with a CL of 130 μm and a GW of 365 μm to fully analyse the electrical characteristics. (**a**) Transfer characteristics of 400 TFTs and a typical output characteristic in the inset, (**b**) threshold voltage distribution, (**c**) mobility distribution, (**d**) log on-off current ratio distribution and (**e**) transconductance distribution.

To display the reproducibility of this R2R gravure system, SWCNT-TFT AMs were printed on a second 15 m PET roll again using freshly formulated inks to adjust the wetting and viscosity. Out of the AMs printed along the 15 m PET roll, we randomly selected two AMs with CLs of 80 and 130 μm (with a GW of 365 μm) and fully characterized them. Their extracted electrical parameters are shown in Figs 4 and 5, respectively. The device yields are 97.5% and 98.7% (Figs 4a and 5a), which are highly similar to the samples in Fig. 3, whereas the average V_th_ variation are 5.12 ± 2.27 V and 0.39 ± 1.13 V, respectively (Figs 4b and 5b) for CLs of 80 and 130 μm respectively. The average mobility values are 0.015 ± 0.010 and 0.027 ± 0.011 cm^2^/Vs, respectively (Figs 4c and 5c), and the average on–off current ratios are 847 ± 672 and 498 ± 399, respectively (Figs 4d and 5d). The mobility was determined using the following equation: *μ*_device_ = (*L/V*_D_*C*_ox_*W)(dI*_d_*/dV*_g_*) = (L/V*_D_*C*_ox_*)(g*_*m*_*/W*), where *C*_*ox*_ is the gate oxide capacitance (7.2 ± 0.3 nF/cm^2^ on average for 130 μm CL and 11.4 ± 0.8 nF/cm^2^ on average for 80 μm CL). The attained average transconductance values are 35.8 ± 9.5 and 29.8 ± 6.6 μS, respectively (Figs 4e and 5e), which are lower than the average values in Fig. 3. The major reason for these results is caused by an inconsistent ink transfer of the low viscous SWCNT ink (>20 cP). From these results, the percentage variation of each electrical parameter is different from the previously reported printed AMs using a roll-to-plate gravure^18^,^19^ because of the different SWCNT and printing mechanisms.

**Figure 6:**
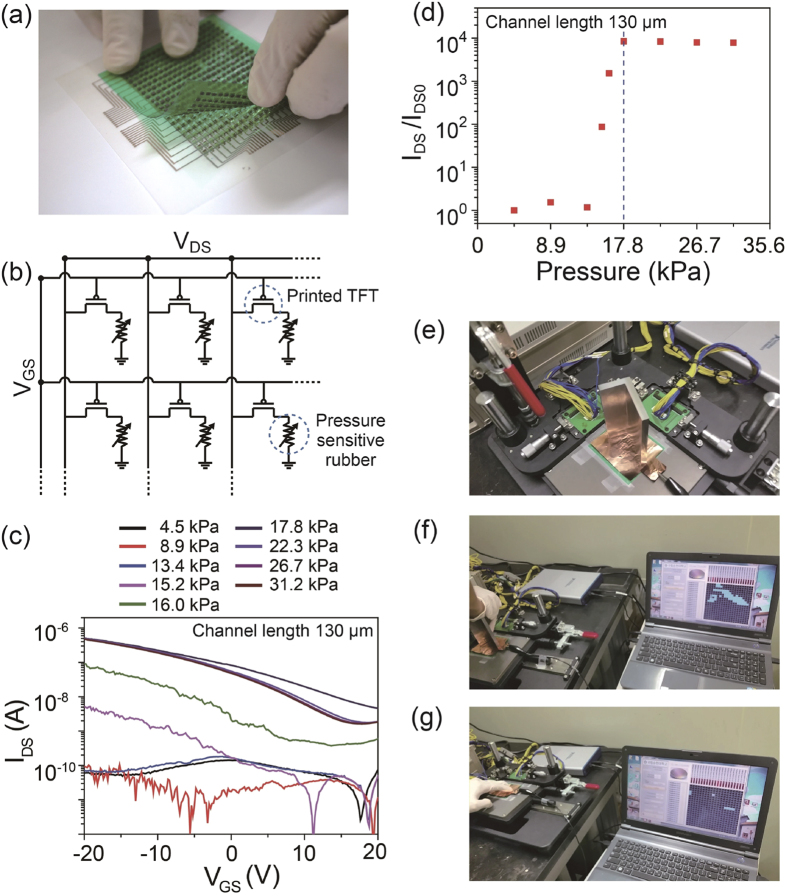
Application of 20 × 20 SWCNT-TFT AM as a tactile sensor. (**a**) Optical image of the PSR laminated on a 20 × 20 TFT AM array with a CL of 130 μm and a GW of 365 μm. (**b**) Equivalent circuit schematic of the pressure sensor array. (**c**) Transfer characteristics of a single pixel under varying pressure loads. (**d**) Measured output current ratio vs. applied pressure with respect to zero applied pressure. (**e,f**) Captured images from video files during the operation of pressure sensor arrays by loading with an “L”-shaped load and (**g**) multi-finger touch (refer a video file for the demonstartion).

Finally, as a proof of concept for the sensor integration and control, a pressure-sensitive rubber (PSR) sheet (CS57-7RSC, PCR Japan) was laminated on a selected 20 × 20 SWCNT-TFT-based AM with a CL of 130 μm and a GW of 365 μm (Fig. 6a). The circuit layout for the pressure sensor application is shown in Fig. 6b. In this pressure sensor array, the source electrodes are in contact with the pressure sensitive rubber, which is connected to a universal ground via a conducting copper sheet. The working concept is to monitor the variations of the source-drain currents caused by the change in the resistance of the laminated rubber, which acts as a pressure-sensitive variable resistor depending on the size of the loading weight when a -20 V gate voltage is applied. To avoid cross-talking in each TFT, we configure the voltage switching module to apply a positive gate voltage ( + 10 V) to the pixels that are not being accessed when the TFT array is being mapped. Based on our setup module (Supplementary Fig. S5), we can simultaneously sense multi-touch points with pressures of more than 17.8 kPa at a speed of 1 Hz. For the actual measurement of each pixel, the current variations of the laminated active matrix are monitored by varying the weights of loads from 4.5 to 31.2 kPa (Fig. 6c,d). Because of the pressure sensitivity of the laminated rubber sheet (CS57-7RSC, PCR Japan), the current in the pixel with a resolution of 9.3 ppi jumps abruptly at a load of 17.8 kPa (Fig. 6d). The same observation is noted when the load is applied to a PSR laminated SWCNT-TFT AM with a CL of 80 μm (Supplementary Fig. S6). Figures 6e–g (see the Supplementary video for a demonstration of the touch sensors) present the results of applying loading weights or finger tactile pressures, on the laminated pressure-sensitive rubber sheet.

## Discussion

Although our group previously reported about printed TFT active matrix using a roll-to-plate gravure^18^,^19^, an R2R gravure is first demonstrated as an advanced manufacturing method to fully print 20 × 20 SWCNT-TFT-based AMs with a 9.3 ppi resolution along a 15 m PET roll by utilizing four rapid and low temperature (7.5 s at 150 °C) curable electronic inks (two silver-nanoparticle-based conducting inks, a BaTiO_3_-nanoparticle-based dielectric ink and a SWCNT-based semiconducting ink). Under this R2R gravure system, we extracted two key scalability factors: the GW and CL. Under the given OPRA of our R2R gravure, adjusting these two factors enables a device yield above 98% while maintaining a constant printing speed, web tension, impression roller pressure, and blading angle. Based on this R2R gravure system, we fabricate AMs with a resolution of 9.3 ppi at a printing speed of 8 m/min. After laminating a PSR sheet on the printed AM, the printed AM can be used as a multi-touch sensing sheet. This design can also be utilized as a surveillance system by monitoring the movement and weight applied across it simultaneously. In the near future, this information can be further modulated to transmit wirelessly to smartphones to maintain long-distance movement surveillance. Furthermore, based on these results, fully R2R gravure-printed signage and displays can be developed by narrowing the range of the V_th_ variation and increasing the resolution through improving the OPRA.

### Methodology

#### Electronic inks

In this study, the silver-nanoparticle-based ink (PG-007, Paru Co., Korea), BaTiO_3_-nanoparticle-based ink (PD-100, Paru Co., Korea), and SWCNT-based ink (PR-040, Paru Co., Korea) were purchased from Paru Co. Korea. Their viscosity and surface tension were adjusted with additives. The details of the resultant viscosity and surface tension characteristics are summarized in Supplementary Table S2.

#### R2R gravure printing

In total, 220 20 × 20 SWCNT-TFT-based AMs were R2R gravure printed on a 15 m PET web with a width of 250 mm and thickness of 100 μm (AH71D, SKC, Korea), using a CCD camera-based registration controller (Supplementary Fig. S7) attached to two printing units (iPen Co. Korea; see Supplementary Fig. S2 for an image of the gravure machine). Six 20 × 20 AMs, with two different GWs (255 and 365 μm), and two different CLs (80 and 130 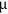
m) can be printed on the PET web by a single rotation of the gravure cylinder with a diameter of 130 mm. To print the four layers of the TFTs completely on the AM using only two printing units, gate lines and dielectric layers are first printed with a printing speed of 8 m/min. Then, the PET films are slowly rewound. During the rewinding process, the printed gate lines and dielectric layers are cured for 1 min by passing through a 150 °C heating chamber. The rewound PET web is printed for a second time. During this phase, the active layers of SWCNTs with an overlay printing registration accuracy of ± 20 μm are printed at the same printing speed of 8 m/min. The SWCNT-printed PET web is rewound, and the source/drain electrodes with an overlay printing registration accuracy of  ±20 μm are then printed. Although we stopped the R2R gravure printing process twice to rewind the PET web, the entire printing process could be performed without interruption using four printing units. The details of the printing conditions are summarized in Supplementary Table S1.

#### Lamination of the PSR sheet

In total, 400 holes with a rectangular shape (1.1 × 1.6 mm^2^) are produced on a PET film with a thickness of 300 μm, by matching the source electrodes of the 20 × 20 active matrix backplanes (AMBs), and then, 400 pieces of rectangular (1.2 × 1.7 mm^2^) PSR (CS57-7RSC, PCR Japan) are packed into the holes of the PET film. The PSR-embedded PET film is laminated on the 20 × 20 AMBs. During the lamination, the alignment accuracy of the 400 embedded PSR pieces on the source electrodes was verified using an optical microscope. A copper foil sheet was then attached to the PSR as a universal ground plane for measurements.

#### Measurements

The surface tension and viscosity of inks were measured using a DCAT21 surface tension meter (Dataphysics Co., Germany) and a SV-10 Vibro viscometer (AND Co., Japan) at a constant temperature of 20 °C. Under ambient conditions, thin film transistors (TFTs) on the AMBs are characterized using a semiconductor parameter analyzer (KEITHLEY 4200, USA). The surface morphology of the printed layers was studied using a surface profiler (NV-220, Nanosystem, Korea) and a microscope (MM6C-DC310-2, Olympus Co. Japan).

#### R2R gravure-printed TFT active matrix-based touch-sensor setup

A custom-made system with a laptop computer (Supplementary Fig. S5) was connected to the gate electrodes and data bus lines (drain electrodes) of the PSR-laminated AM using 20 pins for each connection. Then, DC voltages of -20 and 10 V were periodically applied to each gate electrode, one-by-one, with a periodic rate of 50 ms while a DC of -20 V was applied to the source electrodes. The source/drain currents (I_DS_) of each of the 400 (20 × 20) pixels were measured and converted into digital signals indicating “pressed-on: higher than 2 V (>17.8 kPa)” and “not pressed: lower than 2 V (<17.8 kPa)”. The pixels that output high current levels, indicating applied pressure, were displayed as blue pixels on the laptop screen. The operation of the system was verified by using both an “L”-shaped block and pressures applied via finger pressure.

## Additional Information

**How to cite this article**: Lee, W. *et al.* A fully roll-to-roll gravure-printed carbon nanotube-based active matrix for multi-touch sensors. *Sci. Rep.* **5**, 17707; doi: 10.1038/srep17707 (2015).

## Supplementary Material

Supplementary Information

Supplementary Video 1
