# Supplementary material for: A fully roll-to-roll gravure-printed carbon nanotube-based active matrix for multi-touch sensors
Source: Sci Rep. 2015 Dec 4;5:17707. doi: 10.1038/srep17707 (PMC4669495; doi:10.1038/srep17707)
Supplement: Supplementary Information [file srep17707-s1.pdf]

Fully roll-to-roll gravure printed carbon nanotube based active matrix for multi-touch sensors

Wookyu Lee,[1] Hyunmo Koo,[2] Junfeng Sun,[2] Jinsoo Noh,[2] Kye-Si Kwon,[3] Chiseon Yeom,[2] Younchang Choi,[2] Kevin Chen,[4] Ali Javey[4,]* & Gyoujin Cho[1,2,]*

[1]Regional Innovation Center for Printed Electronics, Sunchon National University, Sunchon 540-742, Korea

[2]Department of Printed Electronics Engineering, Sunchon National University, Sunchon 540-742, Korea

[3]Department of Mechanical Engineering, Soonchunhyang University, Asan 336-745, Korea

[4]Electrical Engineering and Computer Sciences, University of California, Berkeley, California 94720, United States

W. Lee and H. Koo equally share the first authorship.
*Prof. A. Javey and Prof. G. Cho share the corresponding authorship.

Supplementary Information

Table S1. Roll-to-roll gravure printing conditions for printing each layer using a PET web.

	Printing speed (m/min)	printing pressure (kg_f)	Tension (kg_f)	Drying Temperature (℃)	Blading Angle (degree)
Gate electrodes	8	6	5	150	40
Dielectric layers	8	6	5	100	40
Active layers	8	6	5	150	55
Drain-source electrodes	6	6	5	150	40

Table S2. Summary of used ink characteristics for printing 20 × 20 TFT active matrices on 15 m roll of PET using a roll-to-roll gravure.

	Ag ink for gate electrodes	$BaTiO_3$ ink for dielectric layer	SWNT ink for active layer	Ag ink for Source-drain electrodes
Surface tension (mN/m)	48	31.6	25.5	40
Viscosity (cP)	500	100	24	1500
Additives for the formulation	Dipropylene glycol methyl ether	2-Methoxy ethanol	Ethyl acetate	Dipropylene Glycol

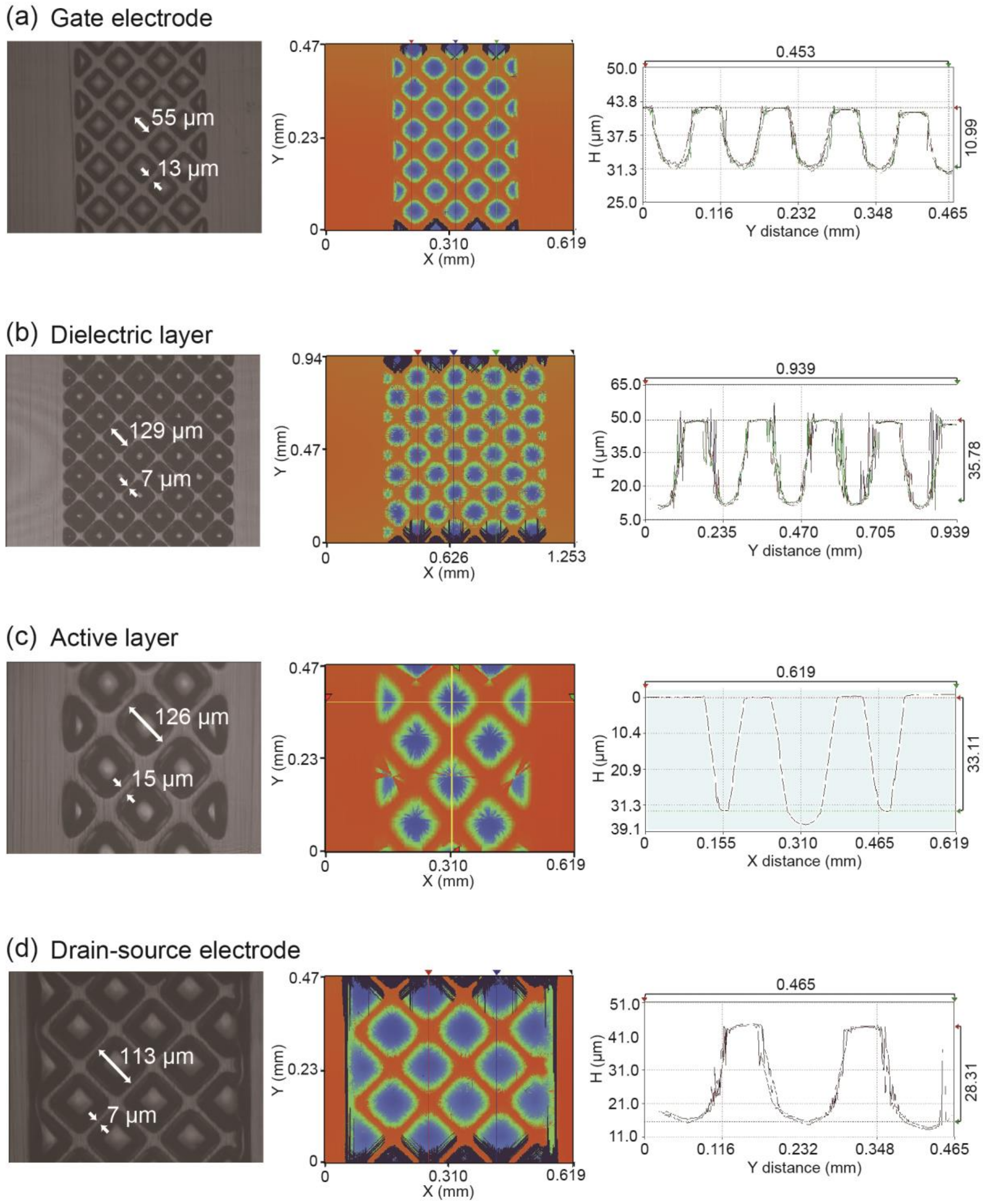

Figure S1. Optical, surface profile, and cross-sectional images of the engraved cell structures on each gravure cylinder used to print the (a) gate, (b) gate dielectric, (c) active, and (d) drain-source electrodes using roll-to-roll gravure

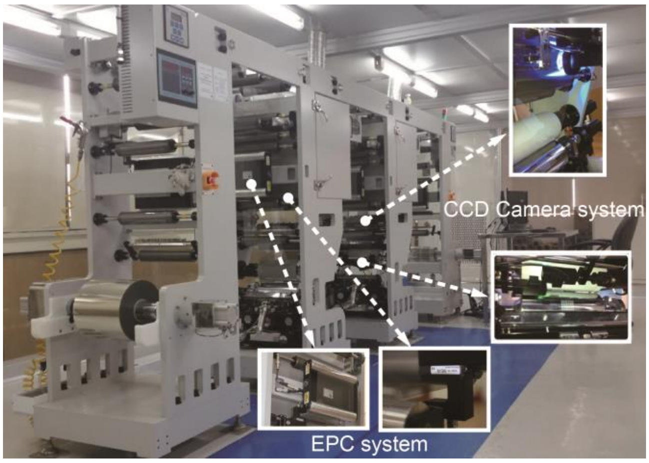

Figure S2. Image of the roll-to-roll gravure machine used in this work. Enlarged images of the EPC and CCD camera system used to control the overlay printing registration accuracy are shown.

(a) Gate electrode width: 365 μm
Channel length: 130 μm

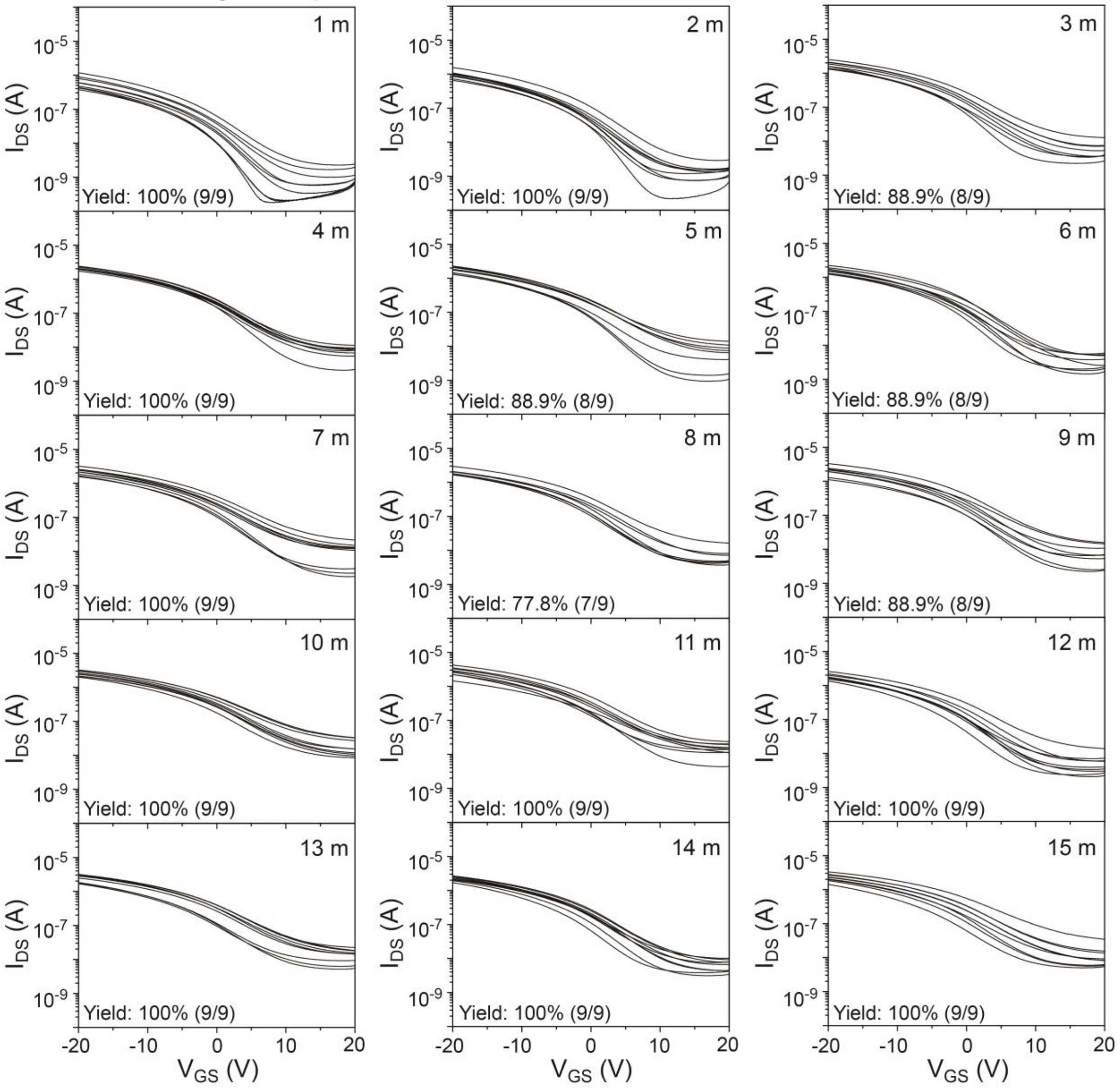

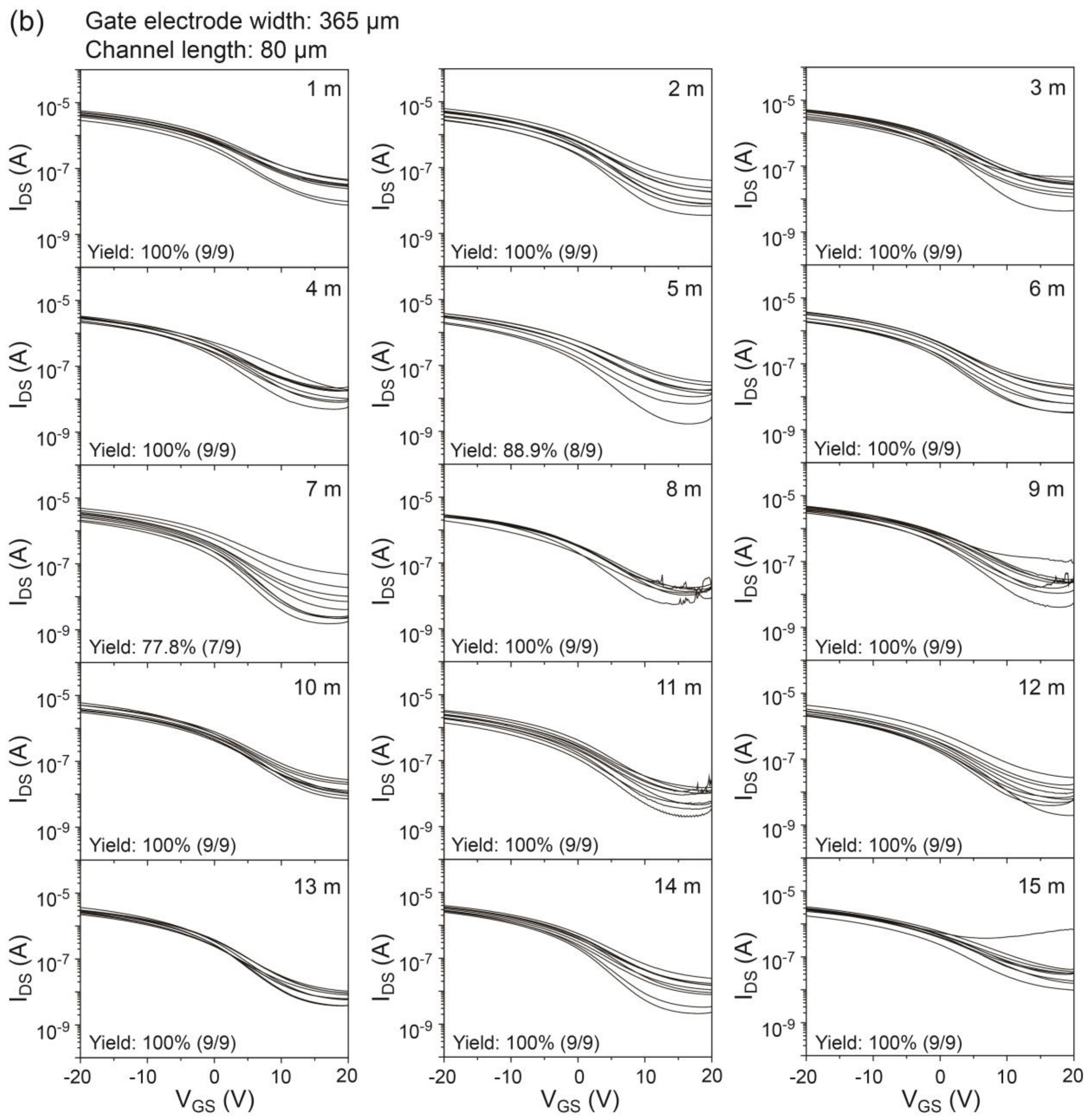

Figure S3. Transfer characteristics of 9 selected TFTs from roll-to-roll gravure printed 20 × 20 TFT active matrices at every 1 m along a 15 m PET roll to show device yield and variation along the length of the PET roll: (a) a channel length of 130 μm at a gate width of 365 μm and (b) a channel length of 80 μm at a gate width of 365 μm.

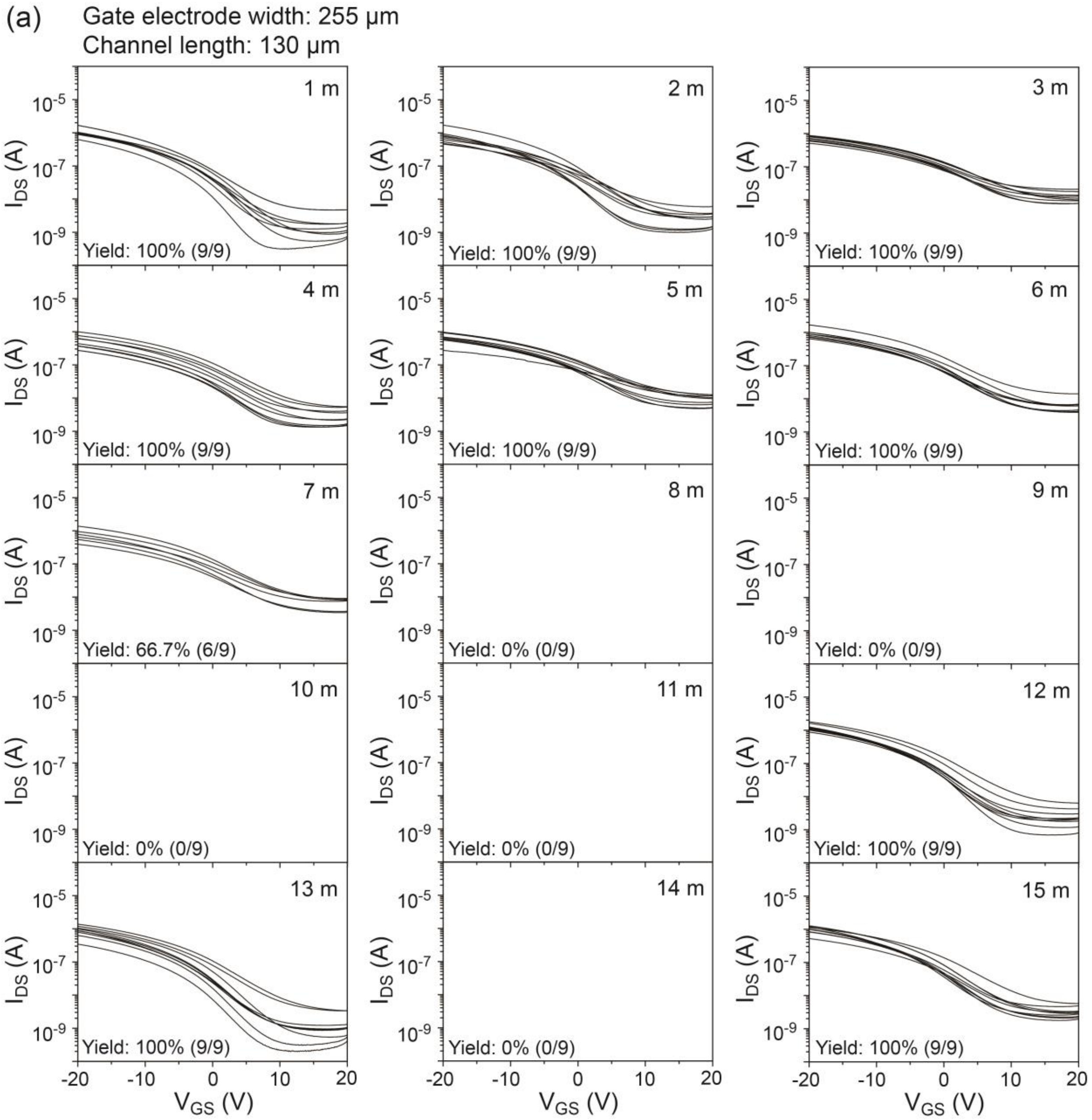
(a)
Gate electrode width: 255 μm
Channel length: 130 μm
1 m
Yield: 100% (9/9)
2 m
Yield: 100% (9/9)
3 m
Yield: 100% (9/9)
4 m
Yield: 100% (9/9)
5 m
Yield: 100% (9/9)
6 m
Yield: 100% (9/9)
7 m
Yield: 66.7% (6/9)
8 m
Yield: 0% (0/9)
9 m
Yield: 0% (0/9)
10 m
Yield: 0% (0/9)
11 m
Yield: 0% (0/9)
12 m
Yield: 100% (9/9)
13 m
Yield: 100% (9/9)
14 m
Yield: 0% (0/9)
15 m
Yield: 100% (9/9)
I_{DS} (A)
V_{GS} (V)

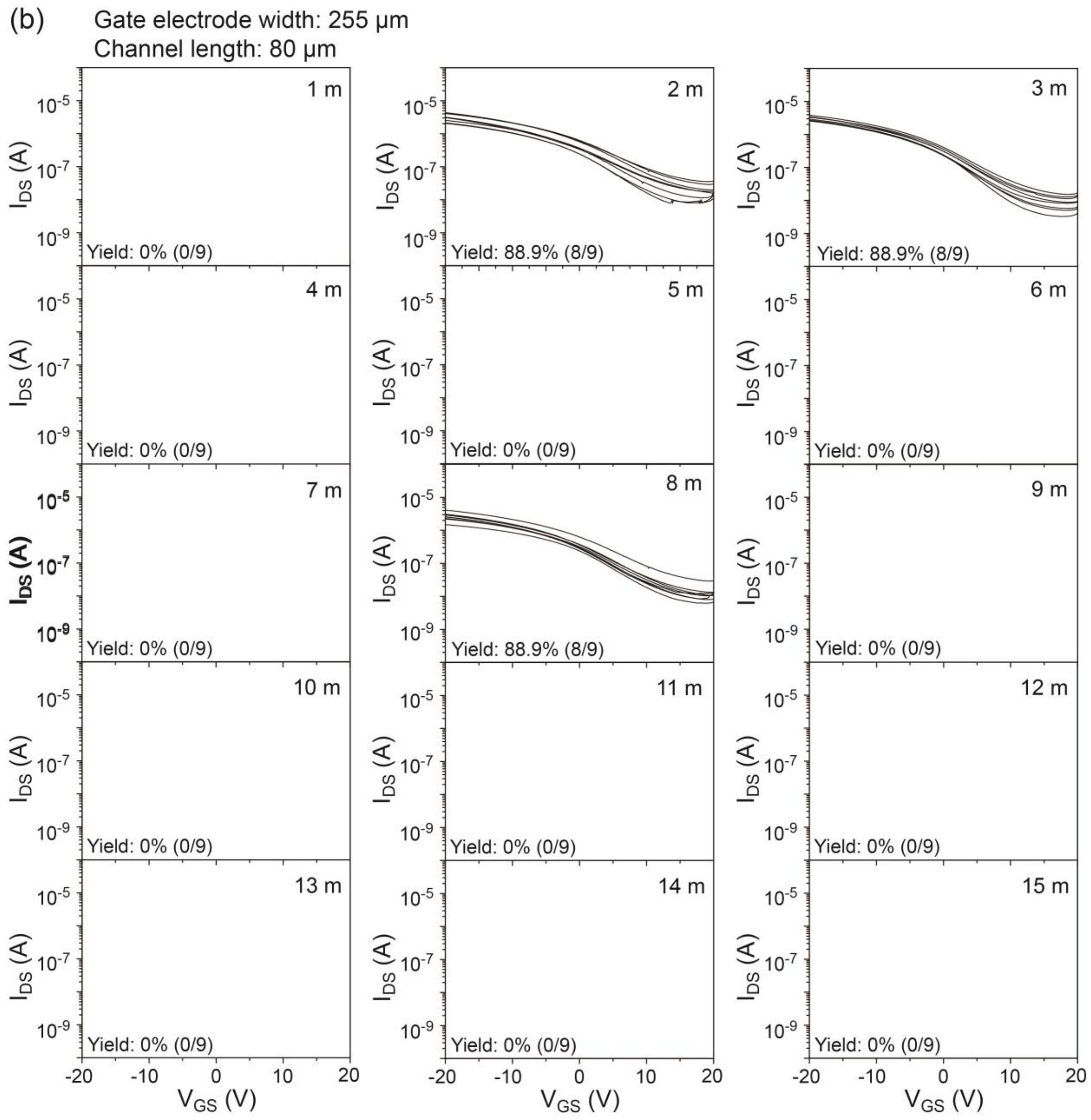

Figure S4. Transfer characteristics of 9 selected TFTs from roll-to-roll gravure printed 20 × 20 TFT active matrices at every 1 m along a 15 m PET roll to show device yield and variation along the length of the PET roll: (a) a channel length of 130 μm at a gate width of 255 μm and (b) a channel length of 80 μm at a gate width of 255 μm.

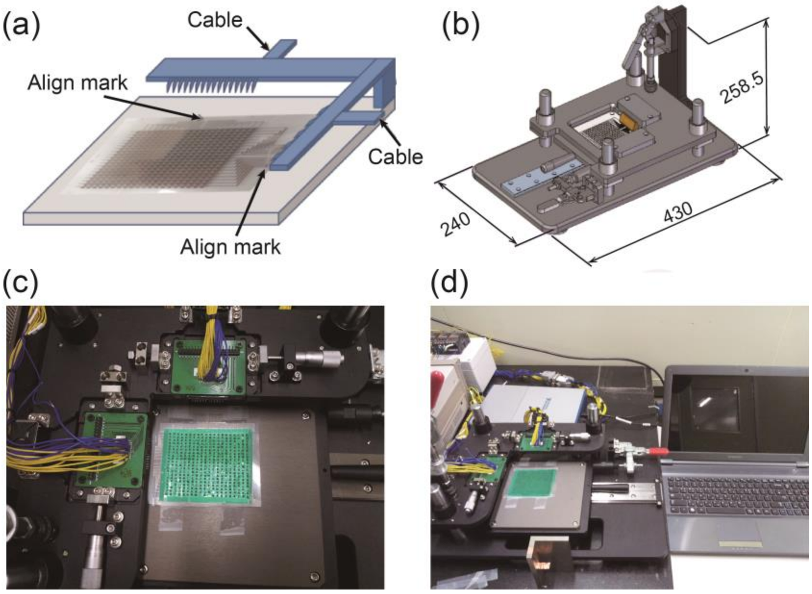

Figure S5. Image of the setup used to monitor the 20 × 20 pressure sensor array in real time.

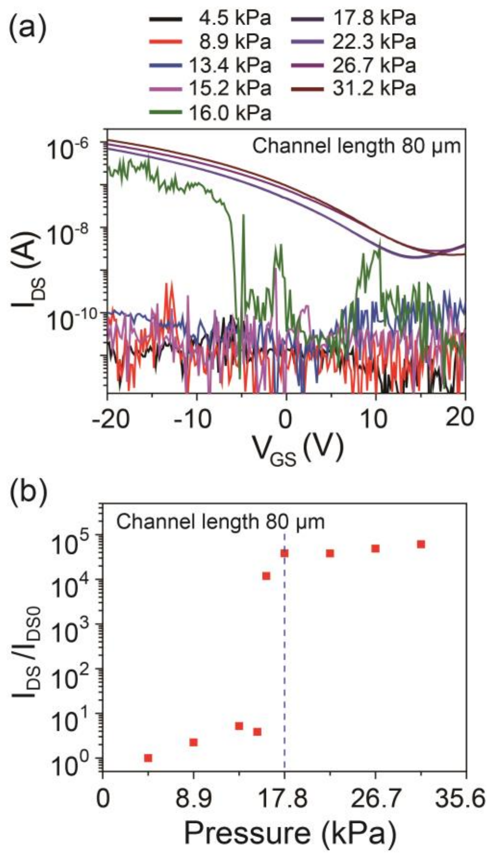

Figure S6. (a) Output transfer characteristics and (b) measured output current ratio with respect to zero applied pressure versus the applied load on a single pixel of the pressure sensor array.

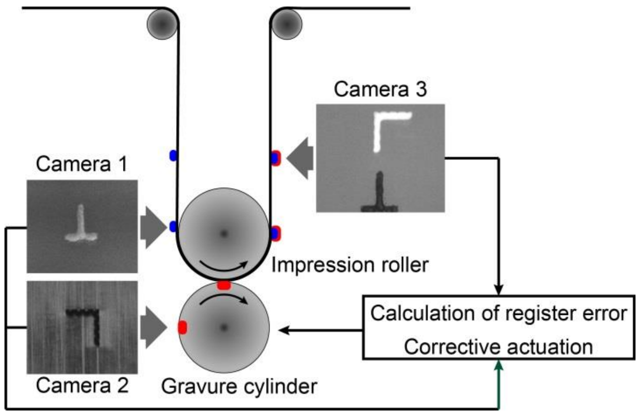

Figure S7. Layout of the servomechanism used to control the overlay printing registration accuracy to ± 20 μm in both the machine and transverse directions. 3 CCD cameras (Figure S2) are used to overcome imperfections in the gravure cylinder. The first CCD camera detects the printed registration marker from the previous the first printing unit. The second CCD camera detects the registration markers on the gravure cylinder at the second printing unit. The mismatch between the attained images of both markers are used to calculate the positional error based on the machine and transverse directions. The errors are amended by slowing down or speeding up the rotation speed of the gravure cylinder as well as fine movements of the gravure cylinder in the lateral direction. Based on this servomechanism, we can attain a registration accuracy of ± 20 μm at a web transfer speed of 8 m/min and actively respond to the expansion of a plastic web (PET ~ -0.6 mm/m) while passing through a 1 m heating chamber (150 ℃) under a web tension of 5 kg_f and the imperfect circumferences of the gravure cylinder rolls.
